# Analysis of risk factors and linear prediction model construction for prolonged mechanical ventilation after Stanford A-type aortic dissection

**DOI:** 10.3389/fsurg.2025.1697977

**Published:** 2025-11-06

**Authors:** Yu Jiajie, Lian Hongmei, Chen Ting, Wei Yanlin, Yali Wang

**Affiliations:** 1 Department of Cardiovascular Surgery of North Sichuan Medical College Hospital, Nanchong, Sichuan, China; 2 Nanchong Health Vocational School, Nanchong, Sichuan, China; 3 School of Nursing, North Sichuan Medical College, Nanchong, Sichuan, China; 4 Affiliated Hospital of North Sichuan Medical College, Nanchong, Sichuan, China

**Keywords:** aortic dissection, mechanical ventilation, predictive models, risk factors, heart surgery

## Abstract

**Objective:**

To explore the risk factors for prolonged acute ventilation time after Stanford type A aortic dissection and to construct a nomogram prediction model.

**Methods:**

A total of 178 patients with Stanford type A aortic dissection admitted to the Department of Cardiac and Vascular Surgery of the Affiliated Hospital of North Sichuan Medical College from 2020 to 2024 were retrospectively enrolled. The patients were randomly divided into a modeling group (124 cases) and a validation group (54 cases) at a 7:3 ratio. Risk factors for prolonged mechanical ventilation time after surgery were analyzed using univariate and multivariate logistic regression analysis, and a risk prediction model was constructed based on the results of multivariate logistic regression analysis.

**Results:**

Multivariate logistic regression analysis showed that age, body mass index, preoperative oxygenation index, cardiopulmonary bypass time, and postoperative serum creatinine were risk factors for prolonged mechanical ventilation time after Stanford type A aortic dissection (*p* < 0.05).A risk prediction model was constructed based on these findings. The area under the ROC curve was 0.91 (95% CI: 0.86–0.97), with an accuracy of 0.88 (95% CI: 0.81–0.93), sensitivity of 0.92 (95%CI: 0.86–0.98), specificity of 0.82 (95%CI: 0.71–0.92), and an optimal cut-off value of 0.527. The results of model validation showed that the area under the ROC curve was 0.79 (95% CI: 0.66–0.92), with an accuracy of 0.72 (95%CI: 0.58–0.84), sensitivity of 0.77 (95%CI: 0.64–0.90), specificity of 0.6 (95%CI: 0.35–0.85).

**Conclusion:**

The prediction model for prolonged mechanical ventilation time in patients with Stanford type A aortic dissection has a good prediction effect and is convenient for clinical use, providing a reference for medical workers to take preventive treatment.

## 1 Introduction

Aortic dissection (AD) is a pathological condition in which blood seeps into the middle layer of the blood vessel wall after the endocardium of the aorta is torn (1). Clinically, the disease is usually classified according to the initial location and extension of the lesion using Stanford classification and Debakey classification. Among them, Stanford type A aortic dissection (TAAD) is an extremely critical type with acute onset and rapid progression (2). Surgical intervention is the main treatment for TAAD patients, but due to the complexity and technical difficulty of the operation, patients often have a variety of postoperative complications, among which prolonged mechanical ventilation time is one of the most common, with an incidence rate of 28.9%–73.3% (3, 4). The prolonged duration of mechanical ventilation will not only reduce the defense capacity of the respiratory system, but also cause diaphragm injury, which may induce lung injury problems such as pulmonary infection and atelectasis, accelerate the deterioration process of the primary disease, and even increase the risk of death during hospitalization by 2.3%–17.0% (5). Based on this, this study aims to construct a prediction model of prolonged postoperative mechanical ventilation time in Stanford type A aortic dissection patients, hoping to provide reference for medical staff to identify high-risk patients early and implement preventive treatment.

## 2 Research subjects and methods

### 2.1 Research subjects

This study retrospectively selected patients with Stanford Type A aortic dissection admitted to the Cardiovascular and Vascular Surgery Department of North Sichuan Medical College Affiliated Hospital from January 2020 to December 2024. The inclusion and exclusion criteria were as follows: Inclusion criteria: (1) Clinically diagnosed as Stanford Type A aortic dissection; (2) surgery for aortic dissection under cardiopulmonary bypass; (3) Complete and reliable medical records. Exclusion criteria: (1) History of mechanical ventilation or pulmonary infection within 14 days prior to surgery; (2) History of organ transplantation, immunosuppressive therapy, or immunodeficiency; (3) Postoperative mortality; (4) Patients discharged without medical supervision; (5) Patients with severe documentation gaps or incomplete clinical data. The study was approved by the Ethics Committee (Approval No.2025ER283-1) and exempted from informed consent. The study adopted the 10EPV rule (6, 7). The study required no fewer than 10 positive events per predictive factor, calculated using the formula *N* = 10*k/p. After reviewing relevant literature, we determined that 3–6 independent variables would be appropriate for the predictive model. Given that postoperative prolonged mechanical ventilation occurs in 28.9% to 73.3% of Stanford A-type aortic dissection cases, a sample size of 41–208 was required (26). This study ultimately enrolled 178 patients.

### 2.2 Collection of clinical data

General data: Gender, age, Body Mass Index (BMI), history of hypertension, diabetes, and smoking. Preoperative data: Time from onset to surgery, white blood cell count, preoperative ischemic stroke, lactate levels, troponin T, D-dimer, serum creatinine, albumin, blood gas oxygenation index, carbon dioxide partial pressure, superimposed C-reactive protein, neutrophil count, pericardial effusion. Intraoperative data: Circulatory time, deep hypothermic circulatory arrest temperature (DHCA temperature), deep hypothermic circulatory arrest time(DHCA time), red blood cell transfusion volume, surgical duration, aortic cross-clamping time, intraoperative blood loss, fresh-frozen plasma transfusion. Postoperative data: Serum creatinine levels, postoperative lactate levels, and postoperative stroke incidence.

### 2.3 Grouping

The definition of prolonged mechanical ventilation time after Stanford type A operation was not clearly stated by many domestic and foreign scholars (8, 9). Based on clinical practice and review of the relevant literature (10, 11), the postoperative mechanical ventilation time of Stanford type A is defined as prolonged if it exceeds 48 h. We defined the postoperative mechanical ventilation time of Stanford type A as prolonged group if it exceeds 48 h and as non-prolonged group if it is less than 48 h.

### 2.4 Statistical methods

The data were processed using SPSS 27.0 statistical software. Count data were expressed as relative numbers, with inter-group comparisons using the *χ*^2^-test. Normal-distributed quantitative data were represented by (*x* ± *s*), with paired *t*-tests for group comparisons. Non-normal distributed quantitative data were presented as M (25,75), with Mann–Whitney *U*-tests for group comparisons. A multi-factor Logistic regression analysis was conducted to explore risk factors for prolonged postoperative mechanical ventilation in acute Stanford A-type aortic dissection patients, followed by model development. The diagnostic efficacy of the model was evaluated through receiver operating characteristic (ROC) curves, while decision curves (DCA curves) were used to assess clinical utility. Significant differences were defined as *P* < 0.05. Categorical variables were coded as follows: gender (1 = female, 2 = male), hypertension (1 = no, 2 = Yes), diabetes (1 = No, 2 = Yes), smoking (1 = No, 2 = Yes), ischemic stroke (1 = No, 2 = Yes), pericardial effusion (1 = No, 2 = Yes), postoperative stroke (1 = No, 2 = Yes).

## 3 Results

### 3.1 Comparison of baseline data

According to the inclusion and exclusion criteria, a total of 178 patients were enrolled in the study, including 108 males and 70 females. Among these patients, 64 cases (35.9%) required prolonged mechanical ventilation duration. The patients were randomly divided into a modeling group (124 cases) and a validation group (54 cases) at a 7:3 ratio. There were no statistically significant difference between the two groups. As shown in Table 1.

**Table 1:** Comparison of basic information between modeling group and validation group.

Project	Total (*n* = 178)	Validation group (*n* = 54)	Modeling modules (*n* = 124)	Statistic	*P*
Body mass index, (Mean ± SD, kg/m^2^)	23.89 ± 2.72	23.85 ± 2.52	23.91 ± 2.81	*t* = −0.13	0.898
Age [M (Q_1_, Q_3_), years]	57.00 (52.00, 66.00)	57.00 (53.00, 65.00)	57.00 (51.00, 66.25)	*Z* = −0.41	0.681
Time from onset to surgery [M (Q_1_, Q_3_), hours]	8.00 (5.00, 12.00)	8.00 (6.00, 12.00)	6.00 (5.00, 12.00)	*Z* = −1.53	0.126
White blood cell count [M (Q_1_, Q_3_),×10^9^/L]	9.93 (8.51, 12.09)	9.10 (8.32, 11.13)	10.24 (8.65, 12.30)	*Z* = −2.71	**0**.**007**
Lactate [M (Q_1_, Q_3_), mmol/L]	1.70 (1.20, 2.50)	1.50 (1.10, 2.55)	1.80 (1.20, 2.50)	*Z* = −1.18	0.239
Cutaneous calcium protein T [M (Q_1_, Q_3_), ug/L]	0.02 (0.01, 0.06)	0.03 (0.01, 0.07)	0.02 (0.01, 0.06)	*Z* = −0.12	0.906
D-dimer [M (Q_1_, Q_3_), mg/L]	5.32 (3.14, 10.83)	5.15 (3.25, 8.84)	5.59 (2.90, 11.18)	*Z* = −0.21	0.832
Preoperative creatinine level [M (Q_1_, Q_3_), μmol/L]	76.00 (66.00, 88.00)	74.90 (65.65, 86.07)	76.00 (66.73, 88.00)	*Z* = −0.39	0.696
Preoperative albumin [M (Q_1_, Q_3_), g/L]	39.20 (37.50, 41.98)	39.15 (37.12, 42.00)	39.30 (37.58, 41.68)	*Z* = −0.07	0.947
Oxygenation index [M (Q_1_, Q_3_), mmHg]	333.33 (285.53, 408.48)	336.36 (282.58, 403.79)	330.87 (287.57, 409.95)	*Z* = −0.07	0.947
Carbon dioxide partial pressure [M (Q_1_, Q_3_), mmHg]	39.00 (36.25, 42.00)	38.50 (36.00, 42.00)	39.00 (37.00, 42.00)	*Z* = −0.85	0.394
Super sensitive C-reactive protein [M(Q_1_, Q_3_), mg/L]	9.99 (2.24, 36.07)	11.91 (2.48, 47.20)	8.98 (1.82, 29.31)	*Z* = −1.08	0.279
Neutrophil count 9M (Q_1_, Q_3_), ×10^9^/L)	8.95 (7.43, 10.90)	8.61 (7.77, 9.97)	9.32 (7.15, 11.37)	*Z* = −0.96	0.337
Time of cardiopulmonary bypass [M (Q_1_, Q_3_), min]	201.00 (179.00, 234.00)	200.00 (180.00, 235.75)	201.00 (178.00, 230.25)	*Z* = −0.13	0.901
DHCA temperature [M (Q_1_, Q_3_), min]	26.00 (26.00, 26.00)	26.00 (25.62, 26.00)	26.00 (26.00, 26.00)	*Z* = −0.18	0.854
DHCA time [M (Q_1_, Q_3_), min]	14.00 (10.00, 18.00)	13.00 (10.00, 18.00)	15.00 (11.00, 18.00)	*Z* = −0.81	0.420
Surgery time [M (Q_1_, Q_3_), min]	400.00 (367.00, 425.00)	401.00 (372.25, 424.50)	400.00 (366.50, 427.00)	*Z* = −0.59	0.554
Time of aortic block [M (Q_1_, Q_3_), min]	128.00 (101.25, 154.00)	129.50 (107.50, 148.75)	128.00 (97.00, 154.50)	*Z* = −0.19	0.853
Blood loss [M (Q_1_, Q_3_), ml]	900.00 (800.00, 1,030.00)	900.00 (800.00, 1,025.00)	900.00 (800.00, 1,035.00)	*Z* = −0.37	0.712
Red blood cell transfusion volume [M (Q_1_, Q_3_), U]	2.50 (2.00, 3.50)	2.50 (2.00, 3.50)	2.25 (2.00, 3.00)	*Z* = −0.35	0.729
Fresh frozen plasma infusion [M (Q_1_, Q_3_), ml]	500.00 (400.00, 600.00)	475.00 (400.00, 600.00)	500.00 (400.00, 600.00)	*Z* = −0.37	0.709
Postoperative creatinine level [M (Q_1_, Q_3_), μmol/L]	106.10 (89.05, 134.28)	102.50 (89.05, 124.20)	109.50 (93.12, 136.35)	*Z* = −1.45	0.148
Gender, *n* (%)				*χ*^2^ = 0.01	0.937
1	70 (39.33)	21 (38.89)	49 (39.52)		
2	108 (60.67)	33 (61.11)	75 (60.48)		
Hypertension, *n* (%)				*χ*^2^ = 1.61	0.205
1	96 (53.93)	33 (61.11)	63 (50.81)		
2	82 (46.07)	21 (38.89)	61 (49.19)		
Diabetes, *n* (%)				*χ*^2^ = 0.00	1.000
1	166 (93.26)	50 (92.59)	116 (93.55)		
2	12 (6.74)	4 (7.41)	8 (6.45)		
Smoke, *n* (%)				*χ*^2^ = 0.03	0.857
1	107 (60.11)	33 (61.11)	74 (59.68)		
2	71 (39.89)	21 (38.89)	50 (40.32)		
Ischemic stroke, *n* (%)				*χ*^2^ = 0.08	0.772
1	172 (96.63)	53 (98.15)	119 (95.97)		
2	6 (3.37)	1 (1.85)	5 (4.03)		
Pericardial effusion, *n* (%)				*χ*^2^ = 0.14	0.708
1	142 (79.78)	44 (81.48)	98 (79.03)		
2	36 (20.22)	10 (18.52)	26 (20.97)		
Postoperative stroke, *n* (%)				–	1.000
1	177 (99.44)	54 (100.00)	123 (99.19)		
2	1 (0.56)	0 (0.00)	1 (0.81)		

Bold values represent statistics with *p* < 0.05, indicating statistical significance.

### 3.2 Single factor analysis of prolonged mechanical ventilation time in Stanford A type aortic dissection patients after surgery

Univariate analysis of the modeling module revealed statistically significant differences (*p* < 0.05) between the prolonged mechanical ventilation group and the non-prolonged group in age, hypertension, body mass index (BMI), white blood cell count, preoperative lactate levels, D-dimer, preoperative oxygenation index, Time of cardiopulmonary bypass, DHCA temperature, DHCA time, Surgery time, fresh frozen plasma infusion, and postoperative creatinine levels. As shown in Table 2.

**Table 2:** Single factor analysis results of prolonged mechanical ventilation time after surgery in Stanford A type aortic dissection patients.

Project	Total (*n* = 124)	Non-prolonged group (*n* = 75)	Pronged group (*n* = 49)	Statistic	*P*
Body mass index, Mean ± SD, kg/m^2^）	23.91 ± 2.81	22.71 ± 2.44	25.75 ± 2.32	*t* = −6.91	**<** **.** **001**
Age [M (Q_1_, Q_3_), years]	57.00 (51.00, 66.25)	55.00 (50.00, 61.50)	64.00 (55.00, 72.00)	*Z* = −3.47	**<** **.** **001**
Time from onset to surgery [M (Q_1_, Q_3_), hours]	6.00 (5.00, 12.00)	7.00 (5.00, 13.00)	6.00 (5.00, 9.00)	*Z* = −1.18	0.238
White blood cell count [M (Q_1_, Q_3_),×10^9^/L]	10.24 (8.65, 12.30)	9.52 (8.51, 11.67)	11.96 (9.99, 12.54)	*Z* = −3.00	**0** **.** **003**
Lactate [M (Q_1_, Q_3_), mmol/L]	1.80 (1.20, 2.50)	1.70 (1.20, 2.35)	2.10 (1.50, 5.20)	*Z* = −2.42	**0** **.** **016**
Cutaneous calcium troponin T [M (Q_1_, Q_3_), ug/L]	0.02 (0.01, 0.06)	0.02 (0.01, 0.06)	0.02 (0.01, 0.09)	*Z* = −0.59	0.555
D-dimer [M (Q_1_, Q_3_), mg/L]	5.59 (2.90, 11.18)	4.36 (2.84, 6.77)	9.32 (3.78, 20.39)	*Z* = −3.46	**<** **.** **001**
Preoperative creatinine level [M (Q_1_, Q_3_), μmol/L]	76.00 (66.73, 88.00)	75.70 (67.00, 84.35)	84.00 (63.50, 107.50)	*Z* = −1.86	0.062
Preoperative albumin [M (Q_1_, Q_3_), g/L]	39.30 (37.58, 41.68)	39.70 (38.00, 42.00)	38.50 (36.70, 41.00)	*Z* = −1.75	0.080
Oxygenation index [M (Q_1_, Q_3_), mmHg]	330.87 (287.57, 409.95)	351.82 (298.49, 421.21)	303.03 (257.58, 372.73)	*Z* = −2.73	**0** **.** **006**
Carbon dioxide partial pressure [M (Q_1_, Q_3_), mmHg]	39.00 (37.00, 42.00)	39.00 (37.00, 41.50)	41.00 (37.00, 43.00)	*Z* = −1.22	0.222
Super sensitive C-reactive protein [M(Q_1_, Q_3_), mg/L]	8.98 (1.82, 29.31)	7.20 (1.67, 32.95)	10.09 (2.13, 21.60)	*Z* = −0.16	0.870
Neutrophil count 9M (Q_1_, Q_3_), ×10^9^**/**L)	9.32 (7.15, 11.37)	8.56 (6.68, 11.45)	9.83 (8.14, 11.35)	*Z* = −1.47	0.142
Time of cardiopulmonary bypass [M (Q_1_, Q_3_), min]	201.00 (178.00, 230.25)	187.00 (173.00, 212.00)	219.00 (195.00, 264.00)	*Z* = −3.53	**<** **.** **001**
DHCA temperature [M (Q_1_, Q_3_), min]	26.00 (26.00, 26.00)	26.00 (26.00, 26.00)	26.00 (24.60, 26.00)	*Z* = −4.01	**<** **.** **001**
DHCA time [M (Q_1_, Q_3_), min]	15.00 (11.00, 18.00)	13.00 (9.00, 17.00)	16.00 (12.00, 20.00)	*Z* = −3.17	**0** **.** **002**
Surgery time [M (Q_1_, Q_3_), min]	400.00 (366.50, 427.00)	391.00 (363.50, 417.00)	408.00 (387.00, 457.00)	*Z* = −2.80	**0** **.** **005**
Time of aortic block [M (Q_1_, Q_3_), min]	128.00 (97.00, 154.50)	127.00 (97.00, 148.00)	147.00 (97.00, 172.00)	*Z* = −1.66	0.098
Blood loss [M (Q_1_, Q_3_), ml]	900.00 (800.00, 1,035.00)	900.00 (810.00, 1,000.00)	1,000.00 (800.00, 1,100.00)	*Z* = −1.91	0.057
Red blood cell transfusion volume [M (Q_1_, Q_3_), U]	2.25 (2.00, 3.00)	2.00 (2.00, 2.50)	3.00 (2.00, 4.00)	*Z* = −1.62	0.105
Fresh frozen plasma infusion [M (Q_1_, Q_3_), ml]	500.00 (400.00, 600.00)	450.00 (400.00, 600.00)	600.00 (400.00, 700.00)	*Z* = −3.79	**<** **.** **001**
Postoperative creatinine level [M (Q_1_, Q_3_), μmol/L]	109.50 (93.12, 136.35)	103.60 (87.90, 119.30)	130.40 (109.00, 189.70)	*Z* = −4.28	**<** **.** **001**
Gender, *n* (%)				*χ*^2^ = 0.02	0.892
1	49 (39.52)	30 (40.00)	19 (38.78)		
2	75 (60.48)	45 (60.00)	30 (61.22)		
Hypertension, *n* (%)				*χ*^2^ = 6.42	**0** **.** **011**
1	63 (50.81)	45 (60.00)	18 (36.73)		
2	61 (49.19)	30 (40.00)	31 (63.27)		
Diabetes, *n* (%)				*χ*^2^ = 0.24	0.621
1	116 (93.55)	69 (92.00)	47 (95.92)		
2	8 (6.45)	6 (8.00)	2 (4.08)		
Smoke, *n* (%)				*χ*^2^ = 0.43	0.510
1	74 (59.68)	43 (57.33)	31 (63.27)		
2	50 (40.32)	32 (42.67)	18 (36.73)		
Ischemic stroke, *n* (%)				*χ*^2^ = 0.24	0.625
1	119 (95.97)	73 (97.33)	46 (93.88)		
2	5 (4.03)	2 (2.67)	3 (6.12)		
Pericardial effusion, *n* (%)				*χ*^2^ = 1.05	0.305
1	98 (79.03)	57 (76.00)	41 (83.67)		
2	26 (20.97)	18 (24.00)	8 (16.33)		
Postoperative stroke, *n* (%)				-	0.395
1	123 (99.19)	75 (100.00)	48 (97.96)		
2	1 (0.81)	0 (0.00)	1 (2.04)		

Bold values represent statistics with *p* < 0.05, indicating statistical significance.

### 3.3 Multivariate regression analysis of prolonged mechanical ventilation in Stanford A-type aortic dissection patients after surgery

We performed a multivariate logistic regression analysis using 13 risk factors that were statistically significant in the univariate analysis as independent variables. The dependent variable was the duration of postoperative mechanical ventilation in patients with Stanford Type A aortic dissection. The model included the following independent variables: age (actual measurement), hypertension (no = 1, yes = 2), body mass index (actual measurement), white blood cell count (actual measurement), preoperative lactate levels (actual measurement), D-dimer (actual measurement), preoperative oxygenation index (actual measurement), Time of cardiopulmonary bypass (actual measurement), DHCA temperature(actual measurement), DHCA time (actual measurement), Surgery time (actual measurement), fresh frozen plasma transfusion (actual measurement), and postoperative creatinine level (actual measurement). The results showed that age, body mass index, preoperative oxygenation index, Time of cardiopulmonary bypass, and postoperative creatinine level were significant predictors of prolonged mechanical ventilation after Stanford Type A aortic dissection (*p* < 0.05), as detailed in Table 3.

**Table 3:** Multivariate regression analysis of prolonged mechanical ventilation time in patients with Stanford A-type aortic dissection after surgery.

Variable	OR	95% CI	*P*
Age	1.10	1.04–1.16	<0.001
Body mass index	1.60	1.28–2.00	<0.001
Time of cardiopulmonary bypass	1.01	1.01–1.02	0.003
Preoperative oxygenation index	0.99	0.99–0.99	0.024
Postoperative creatinine level	1.01	1.01–1.02	0.049

### 3.4 Effect analysis of risk prediction scoring model for prolonged mechanical ventilation time in Stanford A-type aortic dissection patients

**Figure 1:**
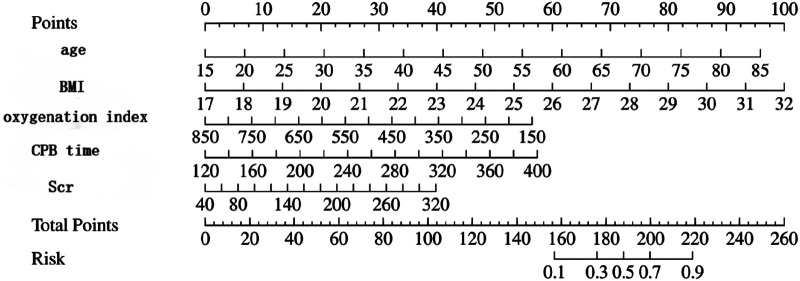
Shows the linear prediction of prolonged mechanical ventilation time after Stanford type A aortic dissection based on patient age, body mass index, preoperative oxygenation index, cardiopulmonary bypass time, and postoperative serum creatinine.

**Figure 2:**
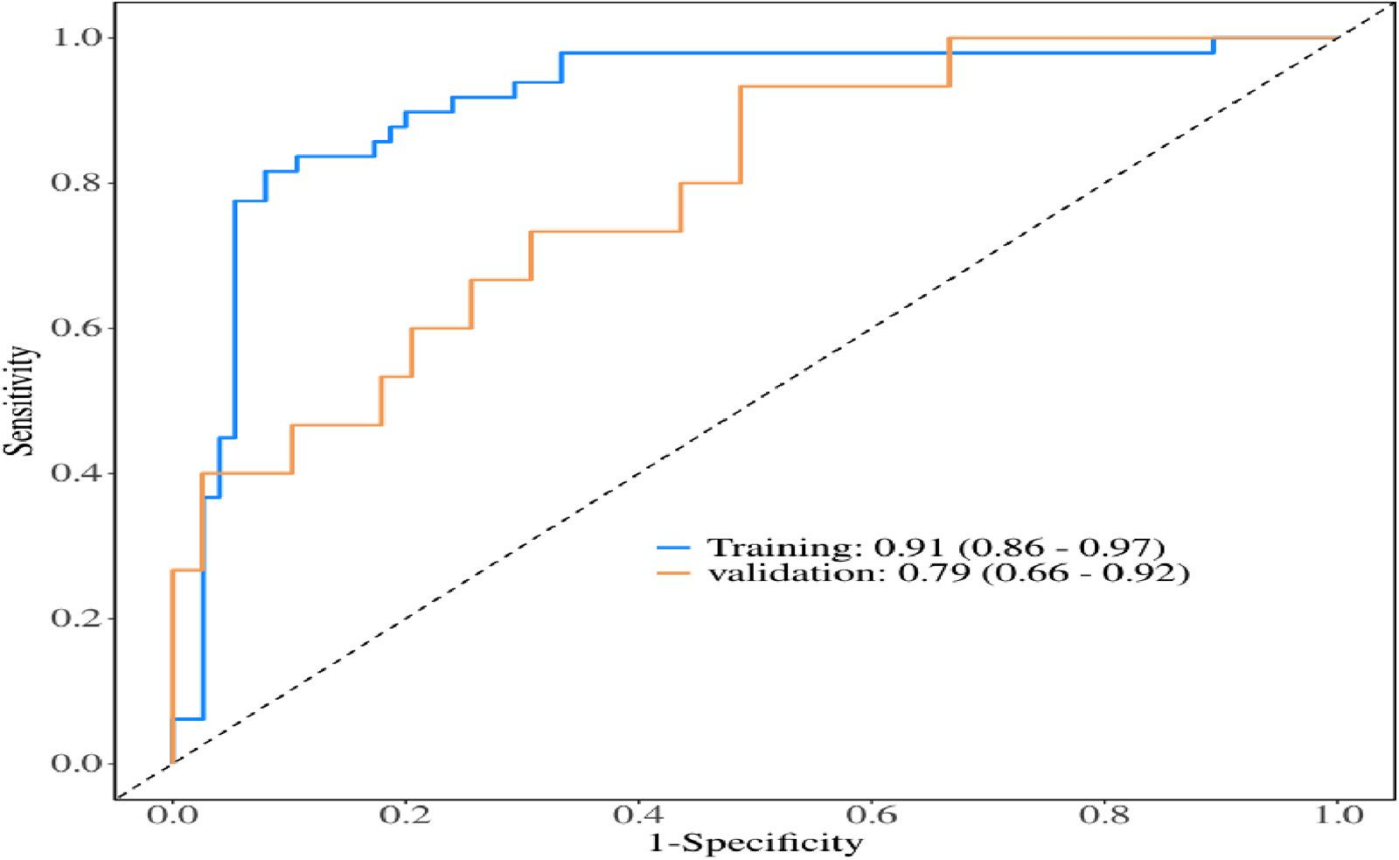
ROC curves of modeling group and verification group.

**Figure 3:**
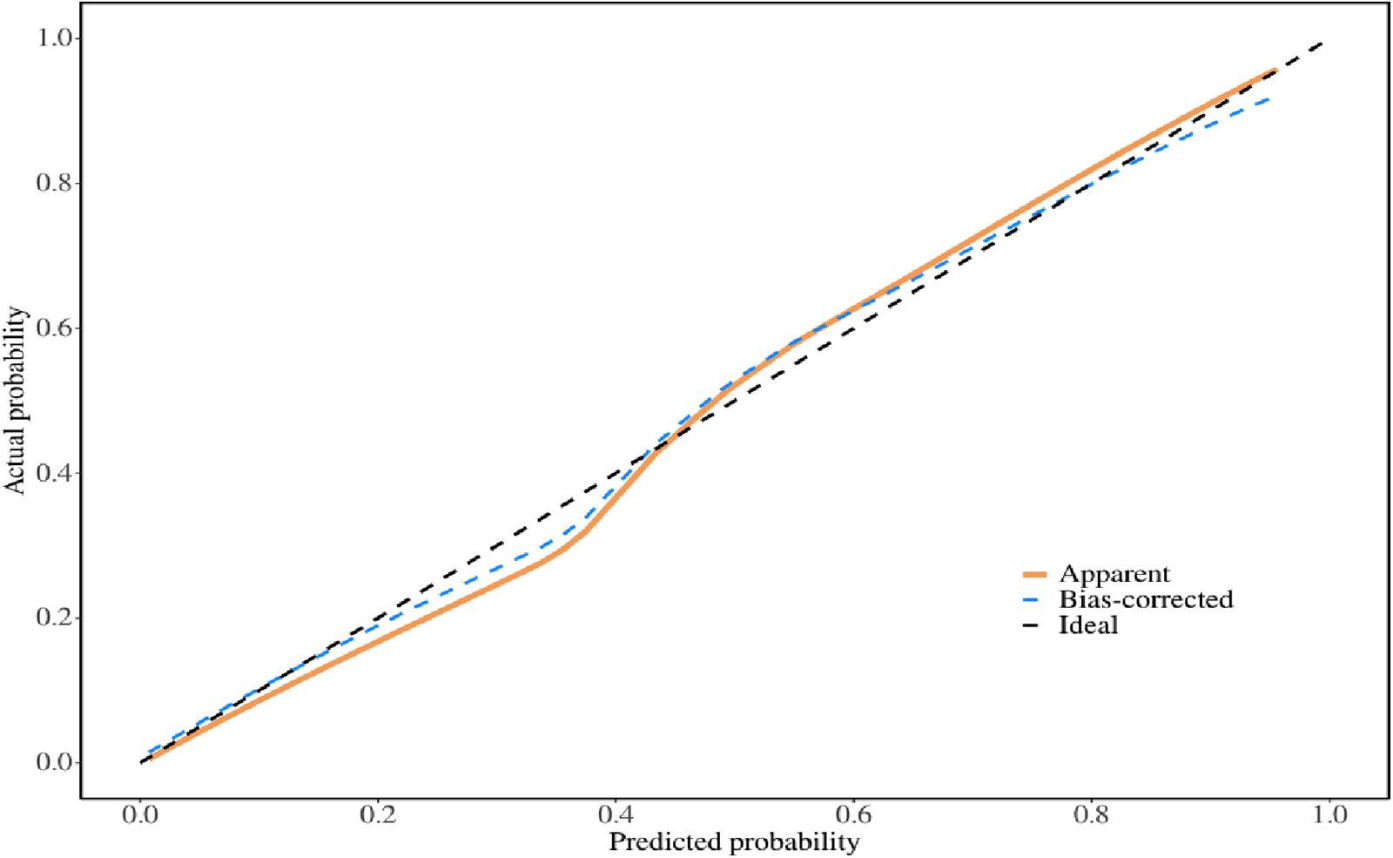
Calibration curve of modeling group.

**Figure 4:**
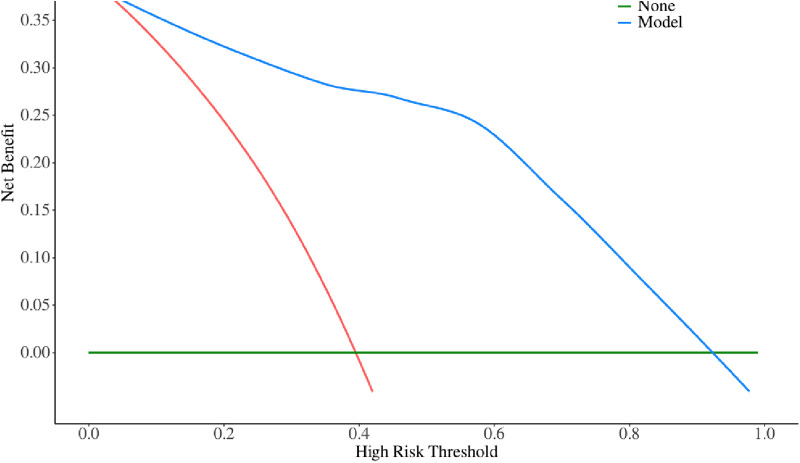
Threshold diagram of modeling group.

The multivariate logistic regression analysis revealed that constructing a predictive model for prolonged mechanical ventilation duration in patients with Stanford Type A aortic dissection, using patient age, body mass index (BMI), preoperative oxygenation index, cardiopulmonary bypass time, and postoperative serum creatinine levels yielded the following results (Figure 1): The model showed an area under the ROC curve (AUC) of 0.91(95% CI: 0.86–0.97 (Figure 2), with accuracy of 0.88 (95%CI: 0.81–0.93), sensitivity of 0.92 (95% CI: 0.86–0.98), and specificity of 0.82 (95% CI: 0.71–0.92). The optimal cutoff value was 0.527 (Table 4). The calibration curve demonstrated a high degree of fit (Figure 3). Model validation results indicated an AUC of 0.79 (95% CI: 0.66–0.92, accuracy of 0.72 (95% CI: 0.58–0.84), sensitivity of 0.77 (95% CI: 0.64–0.90), and specificity of 0.6 (95% CI: 0.35–0.85) (Table 4). Decision curve analysis (DCA) confirmed the models good clinical utility, as illustrated in Figure 4.

**Table 4:** Accuracy, sensitivity, specificity and best cutoff values of the modeling group and the control group.

Model	AUC (95% CI)	Accuracy (95% CI)	Sensitivity (95% CI)	Specificity (95% CI)	PPV (95% CI)	NPV (95% CI)	Cut off
Modeling group	0.91 (0.86–0.97)	0.88 (0.81–0.93)	0.92 (0.86–0.98)	0.82 (0.71–0.92)	0.88 (0.81–0.96)	0.87 (0.77–0.97)	0.527
Validation group	0.79 (0.66–0.92)	0.72 (0.58–0.84)	0.77 (0.64–0.90)	0.60 (0.35–0.85)	0.83 (0.71–0.96)	0.50 (0.27–0.73)	0.527

ppv, positive predictive value; Npv, negative predictive value.

## 4 Discussion

Prolonged postoperative mechanical ventilation duration in patients with Stanford A-type aortic dissection significantly extends hospital stays, exacerbates medical resource consumption, and poses potential threats to both quality of life and patient safety. By developing a predictive model for prolonged postoperative mechanical ventilation, healthcare providers can identify high-risk individuals early and implement targeted management strategies. This approach not only shortens mechanical ventilation duration but also improves surgical outcomes and enhances patients quality of life.

The study found that patient age was associated with the duration of mechanical ventilation after Stanford Type A aortic dissection, which was consistent with the results of Yu Yangtaos team (12). As age increase, cardiopulmonary function gradually declines, and the tolerance to surgical trauma such as cardiopulmonary bypass, decreases. This leads to an increased incidence of respiratory complications and ultimately prolongs mechanical ventilation (13). Elderly patients often present with atypical clinical symptoms, resulting in a high rate of clinical misdiagnosis and diagnostic delay. This not only increases the risk of surgery and patient mortality but also further prolongs the duration of mechanical ventilation and ICU stay (14). Therefore, for this special population of elderly patients, it is crucial to establish a rapid and accurate diagnostic evaluation system and optimize regional medical transfer mechanisms to ensure timely surgical intervention. In postoperative management, maintaining circulatory stability and actively managing chronic underlying pulmonary diseases are equally important. These measures play a significant role in shortening mechanical ventilation duration and improving clinical outcomes. In conclusion, implementing a multidisciplinary collaborative systematic treatment protocol for elderly patients with aortic dissection is a key approach to enhance therapeutic efficacy and reduce complication risks.

This study demonstrates that body mass index (BMI) is a significant risk factor for prolonged postoperative mechanical ventilation duration. In China, obese individuals predominantly exhibit central obesity, characterized by abnormal fat accumulation in the neck, thoracic, and abdominal regions. This adipose tissue deposition restricts respiratory muscle mobility, increases the workload on respiratory muscles, and raises airway resistance. Concurrently, elevated circulating blood volume induces pulmonary congestion, while the increased functional residual capacity under general anesthesia exacerbates alveolar collapse. These combined pathophysiological mechanisms collectively establish the pathological basis for extended mechanical ventilation duration (15, 16). In addition, obese individuals often have long-term chronic inflammation and oxidative stress responses, which activate a variety of inflammatory mediators and signal transduction pathways. These factors cause persistent damage to the lung parenchyma, delay the recovery of respiratory function, and ultimately prolong the time required for ventilation support (17, 18). Therefore, it is of great clinical value to develop individualized perioperative management plans for obese patients. By strengthening respiratory management measures, postoperative mechanical ventilation time can be effectively shortened.

A decreased preoperative oxygenation index is a significant risk factor for prolonged postoperative mechanical ventilation in patients with Stanford Type A aortic dissection. Pathophysiological analysis indicates that reduced preoperative oxygenation typically suggests potential pulmonary dysfunction, inadequate tissue perfusion, and inflammatory responses. Particularly when the dissection affects pulmonary blood supply, this may lead to impaired pulmonary perfusion and substantially diminished gas exchange capacity (19). Other studies have pointed out that (20). Patients with preoperative oxygenation indices below 200 mmHg require 3–5 days longer mechanical ventilation postoperatively compared to those with normal oxygenation levels. Therefore, clinical healthcare providers should proactively implement interventions to improve patients oxygenation status before surgery and strengthen respiratory management after the operation. These measures help reduce complication risks, ultimately shortening mechanical ventilation duration and improving patient outcomes.

This study revealed that Stanford Type A aortic dissection patients with elevated postoperative serum creatinine levels are more likely to require prolonged mechanical ventilation. According to clinical guidelines issued by the Quality of Care Initiative Working Group, once severe acute kidney injury occurs in critically ill patients, the probability of respiratory dysfunction and secondary pulmonary lesions significantly increases (21). From a pathophysiological perspective, this phenomenon is primarily driven by two factors: inflammatory and non-inflammatory components. On one hand, cardiopulmonary bypass surgery may trigger systemic inflammatory responses, leading to altered microvascular permeability and alveolar edema. The subsequent sodium and water retention caused by renal dysfunction further exacerbates this pathological process. On the other hand, patients face dual challenges: a reduced capacity to clear inflammatory factors while experiencing increased production of these substances. Additionally, renal failure weakens immune defense mechanisms and elevates susceptibility to infections (22, 23). These factors combined make patients more prone to lung parenchymal injury, resulting in prolonged use of ventilators.

The findings of this study indicate that prolonged cardiopulmonary bypass (CPB) duration is a significant risk factor for extended postoperative mechanical ventilation in patients with Stanford A-type aortic dissection. CPB procedures performed under non-physiological circulatory conditions notably impair peripheral blood supply and alter capillary permeability, leading to tissue hypoxia and inadequate perfusion. These pathophysiological changes ultimately contribute to pulmonary complications and prolong the required duration of postoperative respiratory support (24). From a pathophysiological perspective, prolonged extracorporeal circulation triggers inflammatory cascades, including complement activation, thrombin generation, and cytokine release. This process also promotes the production of mediators such as endothelin and endotoxins, which impair the function of neutrophils and macrophages, thereby weakening the body's immune defenses. These pathological changes significantly increase the risk of multiple organ dysfunction syndrome (MODS), with the most pronounced damage occurring in vital organs like the lungs, kidneys, and liver (25). In clinical practice, the duration of cardiopulmonary bypass can be effectively controlled by optimizing the surgical plan and improving the technical level of operation, so as to reduce the risk of related complications.

## 5 Summary

This study demonstrates that age, body mass index(BMI), preoperative oxygenation index, cardiopulmonary bypass duration, and postoperative serum creatinine levels are significant predictors of prolonged mechanical ventilation in patients with Stanford Type A aortic dissection. The developed linear model exhibits high predictive accuracy, providing clinical guidance for early identification of high-risk patients requiring extended postoperative ventilation. However, these findings are not consistent with the results of some previous studies. For instance, studies have shown that postoperative blood lactate levels, DHCA time, and elevated preoperative white blood cell counts are associated with prolonged postoperative mechanical ventilation in patients with Stanford Type A aortic dissection, We speculate that it may be related to differences in surgical methods. Our center uses customized stents, which differ from those used in other centers. During the surgery, once the temperature reaches the target, deep hypothermic circulatory arrest with bilateral antegrade cerebral perfusion is performed. The diseased section of the descending aorta is then excised, and the customized intraoperative “elephant trunk” stent is inserted into the descending aorta, immediately restoring circulation. The use of this stent greatly shortens the DHCA time, even reducing it to within 2 min. During the operation, the patient's nasopharyngeal temperature is maintained at 24–28°C. Therefore, in the future, multicenter, large-sample studies are needed to further clarify the related risk factors. Additionally, this model is more suitable for early postoperative risk prediction.

## Data Availability

The data analyzed in this study is subject to the following licenses/restrictions: Concerning the patient's right to privacy. Requests to access these datasets should be directed to 1138703971@qq.com.
